# An Unusual Case of Recurrent Guillain-Barre Syndrome of a Different Subtype Five Years after Initial Diagnosis

**DOI:** 10.1155/2013/356157

**Published:** 2013-04-28

**Authors:** M. Dy, R. L. Leshner, J. R. Crawford

**Affiliations:** ^1^Department of Pediatrics, University of California, San Diego and Rady Children's Hospital, USA; ^2^Departments of Neurosciences and Pediatrics, University of California, San Diego, Division of Child Neurology, Rady Children's Hospital of San Diego, 8010 Frost Street, Suite 400, San Diego, CA 92123, USA

## Abstract

We present a case of a previously healthy 17-year-old girl with history of Guillain-Barre Syndrome 5 years after initial presentation who presented with bilateral lower extremity pain, worsening dysphagia, subsequent weakness, and decreased reflexes. Cerebrospinal fluid analysis had a prominent lymphocytic pleocytosis. MRI of spine showed significant anterior nerve root enhancement. Electromyogram demonstrated a mild axonal greater than demyelinating motor polyneuropathy and intact sensory responses, with no evidence of conduction block or temporal dispersion, unlike her first presentation that revealed a demyelinating polyneuropathy. The patient recovered with mild subjective weakness following 5 days of intravenous immunoglobulin treatment. This case represents a recurrence of a predominantly motor variant polyradiculoneuropathy distinct from the initial presentation with a lymphocytic predominant CSF pleocytosis, nerve root enhancement on MRI spine, and rapid recovery following treatment with intravenous immunoglobulin.

## 1. Introduction

Guillain-Barre Syndrome (GBS) is an immune polyradiculoneuropathy that presents with ascending bilateral lower extremity weakness and areflexia and that affects all age groups with a slight male predisposition [1]. The incidence is 0.89–1.89 cases per 100,000 person-years in Western countries and in severe cases can be fatal [2]. The natural history of GBS in infants and children is more variable and more benign than in adults. Infants may present with hypotonia, feeding difficulties, irritability due to pain, or reduced activity [2, 3]. Limb weakness is both proximal and distal. In 30–45% of pediatric cases, cranial nerves may be more involved, as well as proximal muscles [3]. There could also be slight degrees of motor asymmetry [3]. The most frequent signs and symptoms are paresthesias, weakness, and myalgias [1].

Recurrent Guillain-Barre Syndrome (RGBS) can recur in 1–6% of patients, though it has been reported to occur in 1–10% of patients after asymptomatic period of several months to several years. [3–6] Risk factors for RGBS include age less than 30, milder symptoms, and history of Miller Fisher Syndrome variant [7]. There appears to be no significant difference between RGBS and GBS episodes with respect to similar clinical symptoms and similar or different triggering events. The episode appears to be shorter with half of the patients accumulating deficits [3–5, 7].

We present the case of RGBS of a different subtype 5 years after initial presentation with CSF lymphocytic pleocytosis, nerve root enhancement on MRI, and axonal subtype polyneuropathy with rapid recovery following administration of 5 days of intravenous immunoglobulin. Our case highlights the diverse presentation of RGBS of varied subtype.

## 2. Case Report

A 17-year-old girl, with prior history of GBS, presented to the emergency department with 1 week of bilateral lower extremity pain and 1 day weakness and worsening dysphagia. Her review of systems was remarkable for recent upper respiratory infection. At 12 years of age, she presented with initial episode of GBS, characterized predominantly by pain and dysphagia, which required intubation for rapid progression of symptoms. Her laboratory workup was significant only for mildly elevated creatine kinase and an unremarkable cerebrospinal fluid profile (Table 1). A nerve conduction study during her first presentation demonstrated a primarily demyelinating polyneuropathy with mildly prolonged distal latencies, mildly reduced velocities, temporal dispersion, and preserved sensory responses nonuniformly consistent with both axonal and demyelinating polyneuropathies (Table 2). She received five days of intravenous immunoglobulin (IVIG) and was discharged after 11 days with a normal neurologic examination with exception of 4/5 hip flexor weakness bilaterally.

Her vital signs and general examination at time of her second presentation were unremarkable. Her neurological examination was significant for asymmetric weakness worse on left than on the right in bilateral upper and lower extremities with trace reflexes at ankles and preserved reflexes at patella, biceps, brachioradialis, and triceps in addition to a wide based gait. She had preserved sensory function to light touch, temperature, vibration, and proprioception. Her initial negative inspiratory force (NIF) was at 23 cm H_2_O and was admitted to the intensive care unit. Given her prior medical history of GBS, her symptomatology was consistent with a diagnosis RGBS.

**Figure 1:**
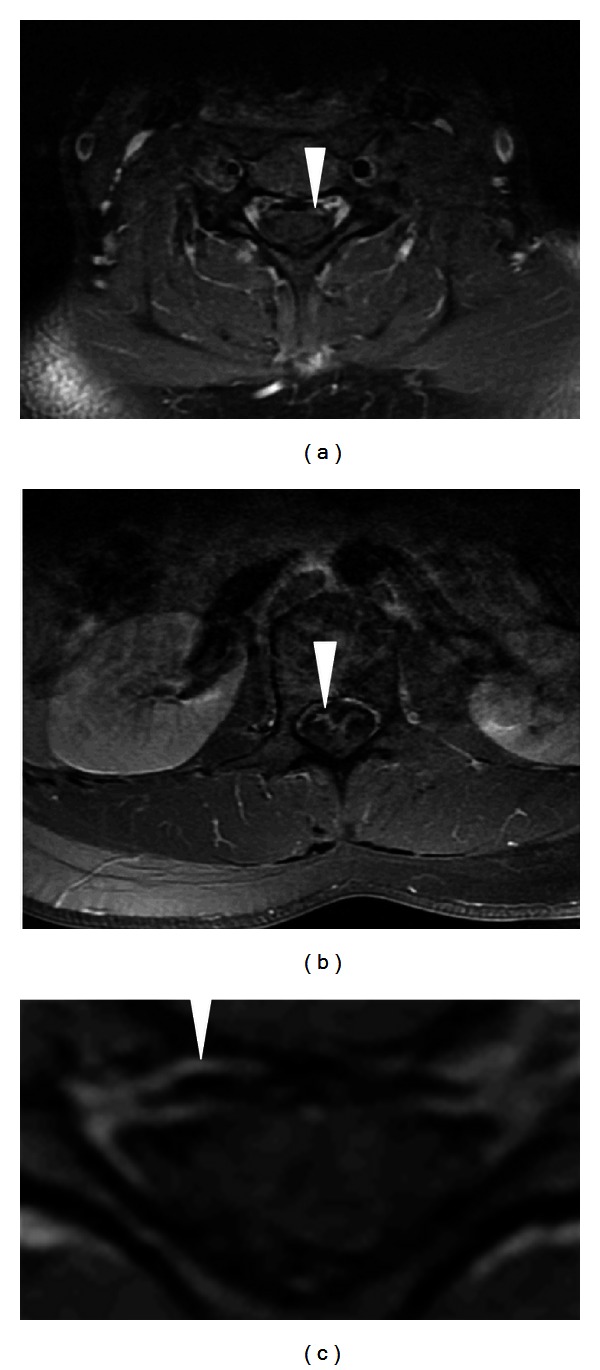
(a) Postgadolinium axial MRI sequences of the cervical cord (a-b), and distal thoracic cord (magnified in (c)) reveals anterior nerve root enhancement consistent with an inflammatory polyneuropathy.

T1-weighted postcontrast fat saturated MRI demonstrated anterior nerve root enhancement of the cervical and lumbar spines (Figure 1). Cerebrospinal fluid was obtained after initiation of IVIG that showed increased protein and a lymphocytic pleocytosis (Table 1). EMG demonstrated mild axonal greater than demyelinating motor polyneuropathy, intact sensory responses, no evidence of denervation on EMG, no evidence of conduction block, and no evidence of temporal dispersion (Table 2). An infectious and rheumatologic workup was nonrevealing, and anti-ganglioside antibodies were negative. She was treated with 5 days of IVIG, with remarkable recovery visible within 24 hours of treatment.

## 3. Discussion

RGBS is a rare entity that has been reported in about 1–6% of all patients with GBS [6]. There are only a few published case studies that include children with RGBS [4, 6, 8, 9]. In these published series patients had both similar and different presentations at recurrence, and many had rapid recovery following therapy. Those patients with multiple recurrences tended to have slower recovery and residual neurologic deficits. The nerve conduction studies tended to show findings similar in patients with monophasic GBS with demyelinating phenotype, with one case report noting that in their population of Japanese patients with RGBS the sensory involvement varied [9]. 

Several perplexing features of our reported case of RGBS include (1) the unusual pattern of weakness at re-presentation, (2) prominent CSF lymphocytic pleocytosis, (3) axonal motor neuropathic phenotype on EMG, (4) dramatic response to IVIG, and (5) MRI findings of contrast enhancement of the anterior cervical and lumbar nerve roots.

The lymphocytic pleocytosis was not typical for GBS or RGBS. This finding expanded the differential diagnosis to include other diagnosis such as infectious, autoimmune, or paraneoplastic polyneuropathies. Furthermore, the EMG/NCS results were not typical of AIDP or CIDP because of early axonal findings as well as the persistence of *F* waves. 

Our patient had a dramatic response to IVG with clinical improvement within twenty-four hours of administration that supports our diagnosis of RGBS. However, it is possible that our patient had an acute motor axonal neuropathy manifesting as initial presentation of chronic relapsing inflammatory polyradiculoneuropathy, given axonal phenotype on EMG. Acute onset chronic inflammatory demyelinating polyneuropathy, which can occur in up to 16% of patients with CIDP with acute onset weakness within 8 weeks, was considered especially in light of the nerve root enhancement on MRI (Figure 1) [8]. This should be a diagnosis of consideration when a patient has deterioration after 9 weeks from onset or when deterioration occurs three times or more. The course may be relapsing remitting, steadily progressive, or monophasic. However, it is more often relapsing or polyphasic than monophasic [1]. Our patient had an axonal motor neuropathy phenotype that does not fit with a diagnosis of CIDP or time course of presentation 5 years after initial diagnosis of GBS. 

One potential explanation of the CSF pleocytosis is that our patient was started on IVIG one day prior to obtaining cerebrospinal fluid. This could have confounded the CSF results and resulted in chemical meningitis, though our patient did not have any meningeal signs. It has been reported that up to about 10% of patients receiving IVIG can develop chemical meningitis depending on disease [10]. Prior cases in patients with Kawasaki disease report that pleocytosis developed within 48 hours. However, the prominent nerve root enhancement on MRI is fully supportive of an inflammatory process such as RGBS. 

To our knowledge, this is the first case of axonal phenotype RGBS in a child. It is important for clinicians to recognize diverse features of RBGS at recurrence. Patients can present with similar symptoms, but have different exam findings, clinical course, and electrodiagnostic studies. RGBS may be an underrecognized and underdiagnosed entity in pediatric patients that is worthy of further study with regard to epidemiology and pathophysiology. 

## Figures and Tables

**Table 1:** Laboratory findings at initial presentation and recurrence of Guillain-Barre syndrome.

Cerebrospinal fluid profile	Initial presentation	Recurrence
Glucose (mg/dL)	54	46
Protein (mg/dL)	22	74
White blood cells	0	46
Red blood cells	1	0
Lymphocytes (%)		74
Neutrophils (%)		2
Monocytes (%)		26

**Table 2:** Nerve conduction/electromyography study on 1st and 2nd presentation of GBS.

Presentation	Nerve	Distal motor latency (DML) ms	Compound muscle action potential amplitude (CMAP) mV	*F* response latency ms	Motor conduction velocity (MCV) m/s	Sensory nerve action potential amplitude uV	Onset latency ms	Peak latency ms
First	Right ulnar	4.3	3.1	No response	49.0			
Second		3.4	(i) at wrist: 2.1 (ii) above and below elbow: 1.5	43	Not performed			

First	Right tibial	10.0	0.3	No response	32.2			
Second		7.0	0.8	56.2	47.3			

First	Right median	3.1	9.0	32.1	58.7			
Second		3.5	3.0	28.4	63.9			

First	Left tibial	5.9	1.1	No response	38.2			
Second		6.2	1.0	54.7	45.6			

First	Right median					91.3	2.7	3.6
Second						99.0	2.3	3.0

First	Right ulnar					53.0	2.1	3.2
Second						69.3	2.0	2.6

First	Right radial					None	None	None
Second						36.2	1.6	2.3

First	Right sural					None	None	None
Second						34.8	2.1	2.7

First	Repetitive nerve stimulation (RNS) of L tibial at a rate of 3 Hz revealed decrement of −3.2 and subsequent increment of 5.5							
Second	None							

## References

[1] Jones HR (1996). Childhood Guillain-Barre syndrome: clinical presentation, diagnosis, and therapy. *Journal of Child Neurology*.

[2] Yuki N, Hartung HP (2012). Guillain-Barré syndrome. *The New England Journal of Medicine*.

[3] Huan M, Smith AG (2012). Weakness, (Guillain-Barré syndrome). *Emergency Neurology*.

[4] Grand’Maison F, Feasby TE, Hahn AF, Koopman WJ (1992). Recurrent guillain-barre syndrome. Clinical and laboratory features. *Brain*.

[5] Hadden RDM (2009). Deterioration after Guillain-Barré syndrome: recurrence, treatment-related fluctuation or CIDP. *The Journal of Neurology, Neurosurgery, and Psychiatry*.

[6] Das A, Kalita J, Misra UK (2004). Recurrent Guillain Barré syndrome. *Electromyography and Clinical Neurophysiology*.

[7] Mossberg N, Nordin M, Movitz C (2012). The recurrent Guillain Barré syndrome: a long-term population-based study. *Acta Neurologica Scandinavica*.

[8] Dionne A, Nicolle MW, Hahn AF (2010). Clinical and electrophysiological parameters distinguishing acute-onset chronic inflammatory demyelinating polyneuropathy from acute inflammatory demyelinating polyneuropathy. *Muscle and Nerve*.

[9] Baba M, Matsunaga M, Narita S (1995). Recurrent Guillain-Barré syndrome in Japan. *Internal Medicine*.

[10] Kemmotsu Y, Nakayama T, Matsuura H (2011). Clinical characteristics of aseptic meningitis induced by intravenous immunoglobulin in patients with Kawasaki disease. *Pediatric Rheumatology Online Journal*.

